# Wool production and quality traits of pure- and crossbred Merino-type sheep

**DOI:** 10.1007/s11250-023-03870-3

**Published:** 2024-01-18

**Authors:** P.G. Theron, T.S. Brand, S.W.P. Cloete, J.H.C van Zyl

**Affiliations:** 1 Department of Animal Sciences, Stellenbosch University, Private Bag X1, Matieland, 7602 South Africa; 2 Directorate: Animal Sciences, Department of Agriculture, Western Cape Government, Private Bag X1, Elsenburg, 7607 South Africa

**Keywords:** Feedlotting, Fibre diameter, Profitability, Terminal crosses, White-wool breeds

## Abstract

Very little information is available on the quality of wool produced by terminal crosses out of wool producing dam lines. This study was therefore undertaken to elucidate the wool quality traits of four crossbred lines relative to Merinos and Dohne Merinos, which served as dam lines. Each dam line was mated to rams of their own breed as well as to Dormer or Ile de France rams to create four crossbred lines, namely, Dohne x Dormer, Dohne x Ile de France, Merino x Dormer, and Merino x Ile de France, in addition to the purebred Dohne Merino and Merino lines. Four rams and four ewes from each of these lines were reared up to one year of age under optimal growth conditions and shorn at the end of that time. Fleeces were weighed and samples collected for analysis. Neither sex nor genotype influenced clean fleece weight or clean yield percentage, but fibre diameter differed between genotypes. Purebred Merino had the finest wool (18.26 μm) and the Merino x Dormer cross the coarsest (26.01 μm). However, all lines still produced wool that could be used in manufacturing clothing, while fleeces showed good uniformity. The two purebred lines had the highest comfort factor (> 98%). The crossbred lines produced a similar quantity of wool as the purebreds, although of poorer quality. All genotypes except Merino x Dormer appear to produce wool that could be processed into garments, although the crossbred wool would only be suitable for outerwear.

## Introduction

According to both the Department of Agriculture, Land Reform and Rural Development (DALRRD 2021) and SA Stud Book (2015), the majority of sheep breeds in South Africa are woolled breeds, most of these being Merino type breeds. During 2019/2020, ~ 43 million tons of wool were sold at auctions, generating R3.75 billion of turnover (CWSA 2020) with the average price per kilogram over all micron classes and lengths being R98.28 for Merino type wool and R54.26 for other wool types (DALRRD 2021). From these statistics, it is clear that wool production plays an important role in the South African sheep industry.

The majority of wool, as mentioned above, is produced by Merino type breeds which include the Merino, Dohne Merino, SA Mutton Merino and Letelle. Wool from these pure breeds are sought after as they generally have low fibre diameters (finer wool), meaning that it is easier to process into high value apparel textiles (Holman and Malau-Aduli 2012; Ferreira et al. 2021). Globally, South Africa is the second largest producer of Merino-type apparel wool and is responsible for 11% of world production (Ferreira et al. 2021). White-wool producing breeds like the Dormer and Ile de France produce carpet wool, which is less sought after than Merino type wool due to its coarseness. A small portion of locally produced wool is also classified as coarse, coloured and Karakul wool (DALRRD 2021) and is generally not very desirable to buyers. In general, South African wool is highly sought after as the South African wool industry is known for producing uniform, high quality wool clips (Ferreira et al. 2021).

Current market demands are centred around fine wool, and the highest prices are paid for wool with a fibre diameter of 17 μm (CWSA 2020) with prices declining as fibre diameter increases. It is therefore to the benefit of producers to produce finer wool. However, wool quality is influenced by a host of factors which in turn impacts on price. These factors include not only fibre diameter but also staple strength, coefficient of variation, comfort factor, spinning fineness, clean fleece yield and fibre curvature, among others (Holman and Malau-Aduli 2012). Next to fibre diameter, staple or tensile strength is considered the most important factor in determining wool quality and therefore price, as it affects the processing quality of wool (Holman and Malau-Aduli 2012; Ferreira et al. 2021). It is measured in Newtons per kilotex (N/ktex) and is defined as the maximum force needed to break a staple (Holman and Malau-Aduli 2012). Since producers are paid per weight of clean wool produced, fleece weight is also an important quantitative trait to consider.

Wool-producing enterprises usually make use of pure breeds with crossbreeding considered to be detrimental to the objectives of wool farmers and being met with scepticism by the wool industry. Owing to the ratio between wool and meat prices as well as improvements in reproduction rate, the bulk of farm income is generated from slaughter lambs. Terminal crossbreeding would allow farmers to utilize heterosis and dimorphism between sire and dam breeds (feeder-breeder dimorphism) (Roux 1992) to improve slaughter lamb production. According to Cloete et al. (2004), the compound effects of heterosis and sexual dimorphism make a commercial terminal crossbreeding system extremely viable even if all replacement ewes are bought in. As a result, some wool producers have turned to crossbreeding with terminal sires to improve farm income and buffer their income stream against fluctuations of the wool market (Cloete et al. 2004; Van der Merwe et al. 2020). The production of wool as a high-value co-product is an additional incentive when Merino-type dams, not suitable for purebred production, are used as terminal dams.

Even in enterprises where the main focus is lamb production, rather than wool, crossbreeding can improve production output markedly (Carneiro et al. 2007; de Sousa et al. 2019). Crossbreeding is therefore a popular practice globally. In South Africa, most crossbred progeny destined for slaughter will be finished off in feedlots or similar intensive finishing systems where profit margins are generally small. In such operations, lambs are frequently shorn just prior to slaughter to generate an extra source of income for the feedlot operator and improve the overall profitability of the enterprise.

While crossbreeding undeniably offers significant benefits to slaughter lamb producers (Sidwell and Miller 1962; Scales et al. 2000; Malhado et al. 2009), the effect of crossbreeding on wool production is less well documented. Therefore, this study aimed to quantify the wool production and quality traits of four crossbred lines relative to purebred Merino (a specialist wool producing breed) and Dohne Merino (a dual-purpose breed) lines as a supplement to the growth modelling of said lines (Theron et al. 2023).

## Materials and methods

Ethical clearance for this study was obtained from Stellenbosch University and was granted under clearance number ACU-2020-14574.

### Animal management

The lambs used in this trial were born on Langgewens Research Farm in the Swartland region of the Western Cape, South Africa, during late autumn and early winter. A mixed flock of Merino and Dohne Merino ewes was randomly divided into three equal groups within breeds, and either mated to rams of their own breeds or to Dormer or Ile de France rams to produce lambs for the trial. Thus, there were six genotypes involved in the trial: Dohne Merino, Dohne x Dormer, Dohne x Ile de France, Merino, Merino x Dormer and Merino x Ile de France. The same rams were used on both dam lines to minimise putative sire effects. The lambs were weaned at approximately 100 days of age and four rams and four ewes of each genotype transported to Elsenburg Research Farm, where they were placed in individual pens (1.5 × 2 m) and reared until one year of age and data collected for a growth modelling exercise that has been published elsewhere (Theron et al. 2023). The lambs were adapted to a commercial feedlot diet (Table 1) over a seven-day period using a step-up program and were allowed *ad libitum* access to this feed for the rest of the trial period. They also had unrestricted access to potable water for the entire period. At the end of the growth period, all animals were shorn by a commercial shearing team and the fleeces collected and weighed. Mid-rib samples were taken from each fleece and sent to the South African Wool Testing Bureau (WTBSA) for analysis and testing. Traits analysed were clean yield, fibre diameter, standard deviation of fibre diameter, coefficient of variation of fibre diameter, comfort factor (the percentage of fibres below 30 μm), staple length and staple strength. The on-farm greasy fleece weight was multiplied with clean yield as a proportion to obtain clean fleece weight.

**Table 1:** Physical and chemical composition on an as fed basis of the pelleted feedlot diet lambs received during the trial

Physical composition	Content (g/kg)	Chemical composition*	Content (%)
Maize meal	500.00	Dry matter	88.86
Lucerne hay	361.00	Ash	4.92
Cottonseed oilcake meal	50.00	Protein	16.38
Molasses powder	25.00	Fibre	7.96
Ammonium chloride	5.00	Fat	2.48
Ammonium sulphate	5.00	Acid detergent fibre	9.01
Lime	5.00	Neutral detergent fibre	18.98
Monocalcium phosphate	5.00	Total digestible nutrients	75.71
Common salt	10.00		
Urea	5.00		
Sodium bicarbonate	10.00		
Slaked lime	5.00		
Sulphur	2.00		
Commercial growth promoters and coccidiostat premix	1.20		
Vitamin and mineral premix	1.50		

*Values derived from proximate analysis of feed (AOAC International 2002)

### Statistical analysis

The results received from the WTBSA as well as clean fleece weight were subjected to a 2 (sex) × 6 (genotypes as described above) factorial design analysis of variance (ANOVA) using Statistica 14 (Tibco Statistica 2020). No adjustments for any traits were made relative to body weight.

Sex and genotype were set as main effects for the analyses, and the possible interactions between these main effects were also considered. Each of the 12 possible treatment combinations was represented by 4 replications. In cases where significant differences were detected between groups at a threshold value of *P* ≤ 0.05, a Fisher’s LSD test was performed post hoc to quantify these differences.

## Results

The results for the main effects of sex and genotype from the ANOVA are given in Table 2 as least squares means. No interaction between sex and genotype occurred for any of the traits considered; thus, the results are presented for the main effects only.

**Table 2:** Various wool quality traits as least squares means (± SE) from yearlings of pure- and crossbred wool type sheep of both sexes

Main effects	Clean fleece weight (kg)	Clean yield (%)	Mean fibre diameter (MFD) (μm)	Coefficient of variation of MFD	Standard deviation of MFD	Comfort factor (%)	Staple length (mm)	Staple strength (N/ktex)
Ram	3.48 ± 0.20	70.03 ± 1.40	23.51 ± 0.31	19.42 ± 0.38^a^	4.59 ± 0.11^a^	85.61 ± 2.62^a^	109.21 ± 2.82	25.42 ± 1.25
Ewe	3.75 ± 0.20	68.71 ± 1.40	22.85 ± 0.30	17.02 ± 0.37^b^	3.92 ± 0.11^b^	93.82 ± 2.55^b^	108.71 ± 2.61	25.54 ± 1.16
*P* value	*0.342*	*0.507*	*0.142*	*< 0.001*	*< 0.001*	*0.030*	*0.897*	*0.942*
Dohne Merino	3.80 ± 0.35	70.48 ± 2.36	20.66^c^ ± 0.52	16.30^c^ ± 0.64	3.38^c^ ± 0.19	98.85^a^ ±4.42	111.00 ± 4.51	29.38^a^ ± 2.01
Dohne x Dormer	3.41 ± 0.35	69.05 ± 2.36	24.53^ab^ ± 0.52	19.43^ab^ ± 0.64	4.78^ab^ ± 0.19	88.50^ab^ ± 4.42	111.25 ± 4.51	25.30^ab^ ± 2.01
Dohne x Ile de France	2.99 ± 0.35	69.81 ± 2.55	24.49^b^ ±0.57	17.98^bc^ ± 0.69	4.43^b^ ± 0.21	80.93^b^ ± 4.78	106.63 ± 5.53	26.63^a^ ± 2.46
Merino	4.31 ± 0.35	71.86 ± 2.36	18.26^d^ ± 0.52	17.69^bc^ ± 0.64	3.24^c^ ± 0.19	99.44^a^ ± 4.42	103.25 ± 4.51	27.63^a^ ± 2.01
Merino x Dormer	3.77 ± 0.35	68.05 ± 2.36	26.01^a^ ± 0.52	20.30^a^ ± 0.64	5.29^a^ ± 0.19	81.74^b^ ± 4.42	113.75 ± 4.51	19.88^b^ ± 2.01
Merino x Ile de France	3.42 ± 0.35	66.96 ± 2.36	25.13^ab^ ± 0.52	17.37^bc^ ± 0.64	4.44^b^ ± 0.19	88.84^ab^ ± 4.42	107.88 ± 4.51	24.13^ab^ ± 2.01
*P* value	*0.173*	*0.740*	*< 0.001*	*0.001*	*< 0.001*	*0.018*	*0.639*	*0.038*

Means with different superscripts (^a–d^) in the same column differ significantly (*P* ≤ 0.05)

Neither clean fleece weight nor the percentage clean yield differed significantly between sexes or genotypes, from which it can be inferred that wool production would be similar regardless of sex or genotype for this experimental outlay. The mean fibre diameter (MFD) differed among genotypes (*P* < 0.001) but not between sexes (*P* = 0.142). Merinos, as expected, had the lowest MFD of 18.26 μm, significantly lower than Dohne Merinos (20.66 μm). In turn, Dohne Merinos had significantly finer wool than Dohne x Ile de France (24.49 μm) which did not differ significantly from the Dohne x Dormer (24.53 μm) and Merino x Ile de France (25.13 μm) groups. The Merino x Dormer cross had the coarsest wool with a MFD of 26.01 μm which was not significantly greater than that of the latter two groups.

The coefficient of variation of fibre diameter and standard deviation of fibre diameter differed significantly between sexes and genotypes. Ewes had a smaller coefficient of variation (17.02%) and standard deviation of fibre diameter (3.92 μm) than rams, indicating a more uniform clip. Dohne Merinos had the lowest coefficient of variation of fibre diameter (16.3%), while purebred Merinos and Dohne Merinos had the lowest standard deviation of fibre diameter of respectively 3.24 μm and 3.38 μm. Thus, purebred Dohne Merinos produced the most uniform clip with regard to fibre diameter. The least uniform clip was given by Merino x Dormer which had a significantly higher coefficient of variation (20.30%) and standard deviation of fibre diameter (5.29 μm) compared to the other genotypes.

Comfort factor, which relates to the degree of possible irritation wearing of a wool garment, can have upon the skin, differed between sexes (*P* = 0.030) and genotypes (*P* = 0.018). Wool from ewes (93.82%) had a higher comfort factor than that from rams (85.61%), while purebred Merinos (99.44%) and Dohne Merinos (98.85%) were the genotypes with the highest comfort factor. These two genotypes did not differ significantly from each other, or from the Dohne x Dormer and Merino x Ile de France crosses. The latter two genotypes did not differ from the Dohne x Ile de France and Merino x Dormer combinations (the groups with the worst absolute comfort factor) either. Staple length was unaffected by sex (*P* = 0.897) and genotype (*P* = 0.639), whereas staple strength was independent of sex (*P* = 0.942), but not genotype (*P* = 0.038). The staple strength of Dohne Merinos (29.38 N/ktex), Merinos (27.63 N/ktex) and Dohne x Ile de France (26.63 N/ktex) did not differ, but exceeded that of the Merino x Dormer cross (19.88 N/ktex; *P* < 0.05). Crosses with Ile de France sires were intermediate and did not differ from the extremes.

## Discussion

The National Wool Growers’ Association (NWGA) is the body in South Africa that is responsible for setting wool quality standards and controlling industry practices to meet international market criteria. To objectively judge the quality of wool produced, the quality traits must not only be compared to previous research but also to NWGA standards (NWGA 2002) to ensure that these are met.

As producers are paid for the quantity of wool produced, fleece weight is important. No significant differences were found between the clean fleece weights of either sex or genetic groups in this study. Clean yield percentages did not differ either. This implies that the quantity of saleable wool produced would not differ significantly between sex or genotype groups, and therefore this would have no influence on profitability.

When comparing fleece weights to values found in previous studies, it was seen that the Merino fleeces were similar in weight to what was reported in previous studies (Snyman et al. 1998; Cloete et al. 2001; Cloete and Cloete 2015), while Dohne Merino fleeces were generally slightly heavier compared to previous studies (Steinhagen 1986; Cloete et al. 1998; Van Wyk et al. 2008; Cloete and Cloete 2015). The clean yield percentages for the two purebred lines are similar to those in the literature (Steinhagen 1986; Cloete et al. 1998, 2003; Cloete and Cloete 2015; Van der Merwe et al. 2020), and any differences can be attributed to environmental differences, with animals in this trial being housed during the rearing period and not maintained on pasture.

Fibre diameter differed between genotypes as expected, with Merinos having the finest wool. The average fibre diameter of 18.26 μm is less than other observations reported in literature with Cloete et al. (2001) (21.9 μm), Cloete et al. (2003) (22.8–23.3 μm) and Cloete and Cloete (2015) (21.6 μm) all observing greater fibre diameters for Merinos. However, both the environmental conditions and the ages of the animals varied between the studies, possibly explaining the difference in the results. In the study of Van der Merwe et al. (2020) where animals were reared in an environment similar to this trial, an average fibre diameter of 19.6 μm was found for Merinos, and Snyman et al. (1998) reported values from 19.8 to 23 μm over three different flocks. It can thus be contended that the present results match existing data.

The average fibre diameter of 20.66 μm found for Dohne Merino in this study compares well to the value of 20.90 μm of Cloete and Cloete (2015), the 21.80 μm of Cloete et al. (2001) and the 21.00 μm of Van der Merwe et al. (2020) while also being close to the 19.36 μm of Van Wyk et al. (2008) on yearling sheep. Matching these results to NWGA standards show that Merino wool would be classified as superfine, while Dohne Merino wool could be regarded as medium-fine. All the crossbred genotypes had fibre diameters in excess of 24 μm and would therefore be classified as overstrong and subsequently have a lower market value. Purebred Dormer and Ile de France breed averages in South Africa have been reported as 27 and 23–27 μm respectively by Snyman (2014a, 2014b). Other reported fibre diameters for yearling Dormers are 31.3 μm (Van der Merwe et al. 2020) and 28.8 μm (Muller et al. 2020). The crosses therefore show improved wool quality when compared to the sire breeds. Although the crossbred groups did not differ significantly from one another, absolute values favoured the two Dohne cross groups even though purebred Merinos had significantly finer wool than purebred Dohne Merinos. This result was unexpected and could possibly be related to sampling, given the relatively small number of animals (*n* = 8) representing each genotype. The increased fibre diameter in crossbred animals was expected, as both white-woolled terminal sire breeds are known to produce coarse wool. In the absence of purebred Dormer and Ile de France progeny, it was impossible to determine heterosis. However, heterosis for wool traits is generally low, and the derived means are unlikely to deviate from the mid-parent value of the pure breeds.

The standard deviation of mean fibre diameter observed for each group in the trial, although differing significantly between both sexes and genotypes, still fell within the guidelines set by the NWGA for each micron class. Thus, all the fleeces displayed satisfactory uniformity according to the standard deviation of mean fibre diameter. The same principle holds true for the coefficient of variation of mean fibre diameter.

The next important factor determining the suitability of wool for different applications is the comfort factor. This trait is related to wearing ease, as wool fibres with a diameter of less than 30 μm are deflected upon contact with the skin and therefore do not cause irritation when worn next to the skin. This makes such wool more suitable for apparel applications (Holman and Malau-Aduli 2012). Both purebred lines exhibited comfort factors in excess of 95%, which is regarded as a threshold for wool worn next to the skin. Similar values were obtained by Van der Merwe et al. (2020). The wool of these traditional wool breeds (Merino and Dohne) would thus be suitable for processing into knitwear or underwear based on its fineness and wearing ease (NWGA 2002). The Merino x Dormer and Dohne x Ile de France crosses displayed lower (*P* < 0.05) comfort factor values. Wool from these crosses will not be as sought after, although still suitable for processing into outerwear like heavy overcoats (NWGA 2002).

Staple length did not differ between the various groups, and all lengths exceeded 90 mm, therefore being classed as AA according to NWGA classification standards (NWGA 2002). The staple lengths of the purebreds were higher than those reported by Cloete and Cloete (2015), who found 89.90 and 90.10 mm as staple lengths for reproducing Merino and Dohne Merino ewes respectively. Contrastingly, the values were much lower than the 158.97 mm for Merinos and 141.67 mm for Dohne Merinos reported by Van der Merwe et al. (2020) but almost identical to the value of 111 mm reported for Dohne Merinos by Cloete et al. (1998). The reason for the variation between studies may be attributed to Cloete and Cloete (2015) working on reproducing ewes. It is feasible that the metabolic requirements of pregnancy and lactation may have curbed wool growth, resulting in lower staple lengths. Regardless of the reason for the variation, it is clear that staple length varies widely between studies and precise comparison between studies therefore proved to be difficult. At this point, the small sample size used in the current study could also be expected to potentially play a role.

Holman and Malau-Aduli (2012) described staples with a breaking force of 25–30 N/ktex as “sound”, while the NWGA (2002) defines such staples as being of average tensile strength and only rate staples with greater values than 30 N/ktex as being “sound”. The Merino x Dormer cross, with a staple strength of 19.88 N/ktex, is classified as “tender” by both systems, while Merino x Ile de France also falls below the 25 N/ktex threshold. Purebred Merinos and all Dohne Merino lines displayed staple strength values above this threshold and will therefore be more valuable according to Holman and Malau-Aduli (2012). All genotypes however produced wool with a staple strength below 30 N/ktex, which is considered partially tender according to NWGA guidelines (NWGA 2002). It is unclear what the reason for this could be since the animals were under no nutritional or health stress that may have compromised wool quality.

Cloete and Cloete (2015) reported staple strength values of 36.2 N/ktex and 35.1 N/ktex for Merinos and Dohne Merinos respectively, which is higher than the values obtained in this study.

## Conclusion

Overall, it seems that all the genotypes produce wool that is suitable to be processed into clothing, meaning that it falls into the highest value bracket of wool production. Wool from the two specialist wool breeds were suitable to produce garments worn next to the skin, but the wool from all crossbred lines were suitable only for outerwear. This result was expected, as both the Dormer and Ile de France used as terminal sire breeds traditionally have coarse wool (> 27 μm). The quantity of wool produced in this trial did not differ between genotypes, while the mean uniformity of all breed combinations generally conformed to NWGA guidelines. This would improve the potential value of the clip from these animals. Furthermore all groups, with the exception of Merino x Dormer, produced wool that was suitable for processing. The Merino x Dormer cross produced the weakest wool, despite having the greatest fibre diameter, making it less suitable for processing.

Feedlot operators buying in older (> 6 months) crossbred lambs sired by white-woolled terminal sire breeds can therefore still expect reasonable wool quality and quantity from the crossbred offspring, rendering shearing prior to slaughter a potentially profitable intervention.

## Data Availability

The data sets generated during the current study are available from the corresponding author upon reasonable request.
